# Effect of external fixation rod coupling in computed tomography

**DOI:** 10.1007/s11751-018-0318-x

**Published:** 2018-09-15

**Authors:** Carlos A. Peña-Solórzano, Matthew R. Dimmock, David W. Albrecht, David M. Paganin, Richard B. Bassed, Mitzi Klein, Peter C. Harris

**Affiliations:** 1 Department of Medical Imaging and Radiation Sciences, Monash University, Wellington Rd, Clayton, Melbourne, VIC 3800 Australia; 2 Clayton School of Information Technology, Monash University, Wellington Rd, Clayton, Melbourne, VIC 3800 Australia; 3 School of Physics and Astronomy, Monash University, Wellington Rd, Clayton, Melbourne, VIC 3800 Australia; 4 Victorian Institute of Forensic Medicine, 57-83 Kavanagh St., Southbank, Melbourne, VIC 3006 Australia; 5 Department of Forensic Medicine, Monash University, Wellington Rd, Clayton, Melbourne, VIC 3800 Australia; 6 Australian Synchrotron, 800 Blackburn Rd, Clayton, Melbourne, VIC 3168 Australia; 7 The Royal Children’s Hospital Melbourne, 50 Flemington Road, Parkville, Melbourne, VIC 3052 Australia; 8 Department of Orthopaedic Surgery, Western Health, Footscray Hospital, Gordon St, Footscray, Melbourne, VIC 3011 Australia

**Keywords:** Computed tomography, Rod coupling, Dual-energy CT, Metal artefact reduction, Metal artefacts

## Abstract

External fixation is a common tool in the treatment of complex fractures, correction of limb deformity, and salvage arthrodesis. These devices typically incorporate radio-opaque metal rods/struts connected at varying distances and orientations between rings. Whilst the predominant imaging modality is plain film radiology, computed tomography (CT) may be performed in order for the surgeon to make a more confident clinical decision (e.g. timing of frame removal, assessment of degree of arthrodesis). We used a fractured sheep leg to systematically assess CT imaging performance with a Discovery CT750 HD CT scanner (GE Healthcare) to show how rod coupling in both traditional Ilizarov and hexapod frames distorts images. We also investigated the role of dual-energy CT (DECT) and metal artefact reduction software (MARS) on the visualisation of the fractured leg. Whilst mechanical reasons predominantly dictate the rod/strut configurations when building a circular frame, rod coupling in CT can be minimised. Firstly, ideally, all or all but one rod can be removed during imaging resulting in no rod coupling. If this is not possible, strategies for configuring the rods to minimise the effect of the rod coupling on the region of interest are demonstrated, e.g., in the case of a four-rod construct, switching the two anterior rods to a more central single one will achieve this goal without particularly jeopardising mechanical strength for a short period. It is also shown that the addition of DECT and MARS results in a reduction of artefacts, but also affects tissue and bone differentiation.

## Introduction

Circular external fixation is one of several tools available for the orthopaedic surgeon to use in the management of a number of orthopaedic conditions [1], including the treatment of complex fractures, correction of limb deformity, and salvage arthrodesis.

During the post-operative period, imaging is performed to assess, amongst other things, alignment and progress of healing (callus formation, union, etc.). Whilst the predominant imaging modality is plain film radiology, computed tomography (CT) may be performed when plain film radiology does not give sufficient detail of a region of interest (ROI) in order for the surgeon to make a more confident clinical decision (e.g. timing of frame removal, assessment of degree of arthrodesis).

The frame components, being relatively radio-opaque, have the capacity to either obscure the view of the region of interest (predominantly in the case of plain film radiology) or distort the quality of the image to some degree (in the case of CT). For both of these imaging modalities, strategies to minimise this fall under three general categories. Firstly, the surgeon has some degree of choice as to the positioning of certain components (e.g. rings and rods) relative to the region of interest. Secondly, the way in which the patient is positioned during image acquisition can be optimised [2]. Thirdly, some components, such as the connecting rods, can be temporarily removed or repositioned during imaging. With CT there is an additional strategy; many modern scanners have the facility to reduce the effect of metal artefacts through the use of dual-energy CT (DECT) and/or metal artefact reduction algorithms [3–6]. However, such scanner settings are not specific to the individual patient/frame construct, and so not only do they have varying ability to reduce metallic artefacts, but in addition they may also adversely affect image quality in general.

Since there are situations in which the orthopaedic surgeon may rely heavily on the quality of the CT image, this study focuses on the nature of the adverse effect of metal rods/struts on the quality of the image and on how to optimise the image quality in the presence of metallic components.

## Materials and methods

The leg of a sheep that had been euthanised for reasons unrelated to this research was used. A mid-shaft oblique fracture was created by making a single drill hole followed by the use of an osteotome whilst bending the bone. The soft tissues were left in place.

**Fig. 1:**
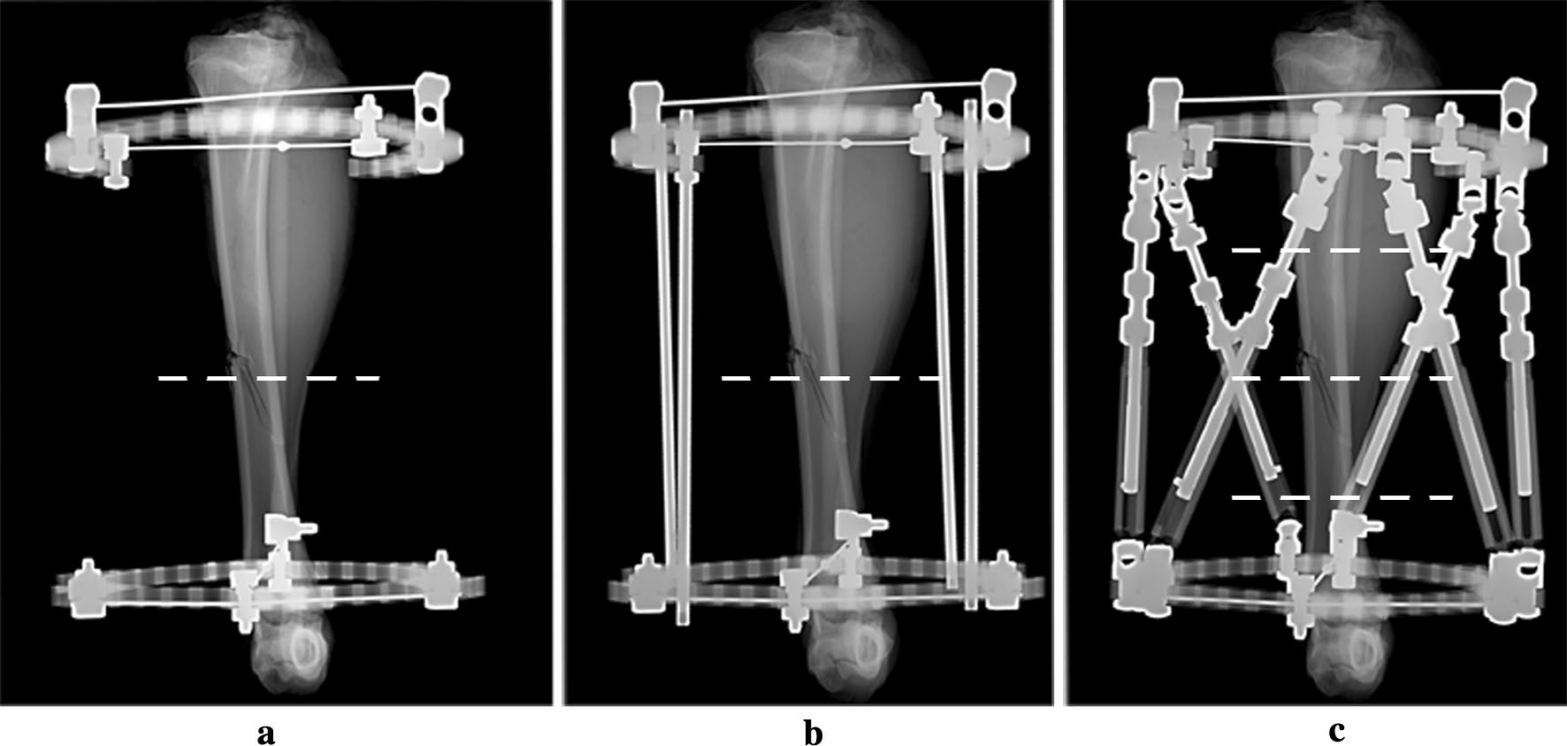
X-rays (anterior–posterior views) showing various frame constructs. **a** Initial positioning of the rings. **b** Ilizarov frame (4 rods). **c** Taylor Spatial Frame. Dotted lines show the position of the slices used for the study

**Fig. 2:**
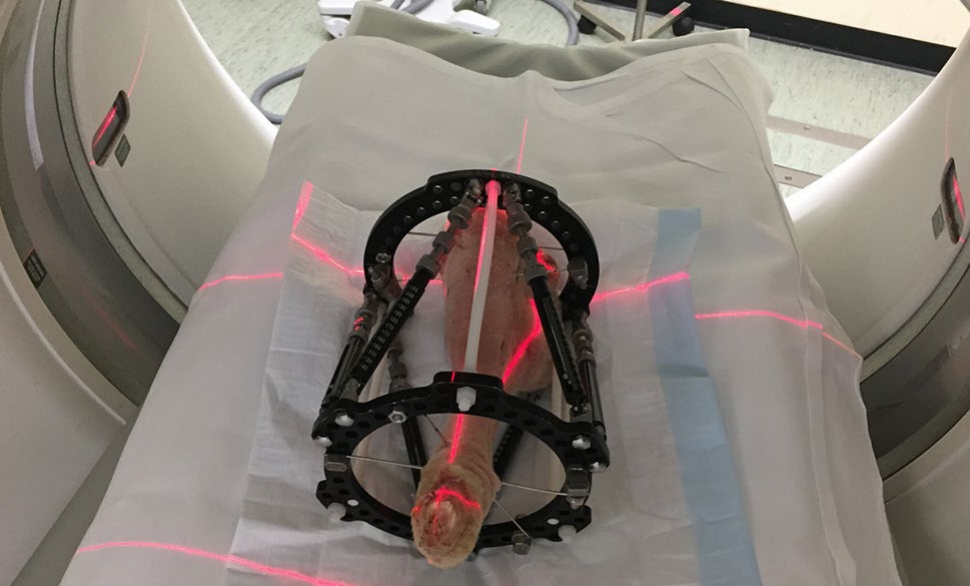
Photograph of the frame/leg construct in the CT scanner

The frame consisted of two 130-mm aluminium rings (Taylor Spatial Frame, Smith & Nephew), spaced 196 mm apart. Two construct types were studied. Construct one represents the traditional Ilizarov frame, where the rings are connected by threaded stainless steel rods (Ilizarov, Smith & Nephew) (see Fig. 1b). Construct two is a hexapod, where the rings are connected by six struts attached at the outer mounting holes of the tabs (Taylor Spatial Frame, Smith & Nephew) (see Fig. 1c). For the Ilizarov frame, the rods were varied both in number and in configuration, whilst for the Taylor Spatial Frame (TSF), only one configuration was used, with all 6 struts at the same length. Each ring was attached to the bone using two crossed and tensioned wires. The rings were connected with three plastic threaded rods, which allowed a baseline scan to be performed without any metal between the rings whilst ensuring that the fracture position remained unchanged. The frame was taped to the CT bed in an orthogonal orientation, ensuring that a constant and idealised position was maintained for all study images. To further ensure standardisation of slice acquisition, the proximal ring was used as a reference level for each axial slice (Fig. 2).

The scans were performed with a Discovery CT750 HD CT scanner (GE Healthcare), which has the facility for both DECT and metal artefact reduction software (MARS). The frame/leg construct was scanned initially at 100 kVp, which represents the single-energy setting that would typically be used in the presence of metal. It was then scanned at dual energy (80/140 kVp) with and without MARS. A single axial slice is composed of $512 \times 512$ (262,144) pixels, whilst the field of view chosen for this study resulted in one pixel being equivalent to 0.39 mm × 0.39 mm of scanned area. Each individual pixel is a measure of relative radiodensity and is given a value termed a Hounsfield unit (HU). Water (at standard pressure and temperature) has a HU of zero, whilst anything of lesser radiodensity is a negative value and anything of greater radiodensity is a positive value [7].

**Fig. 3:**
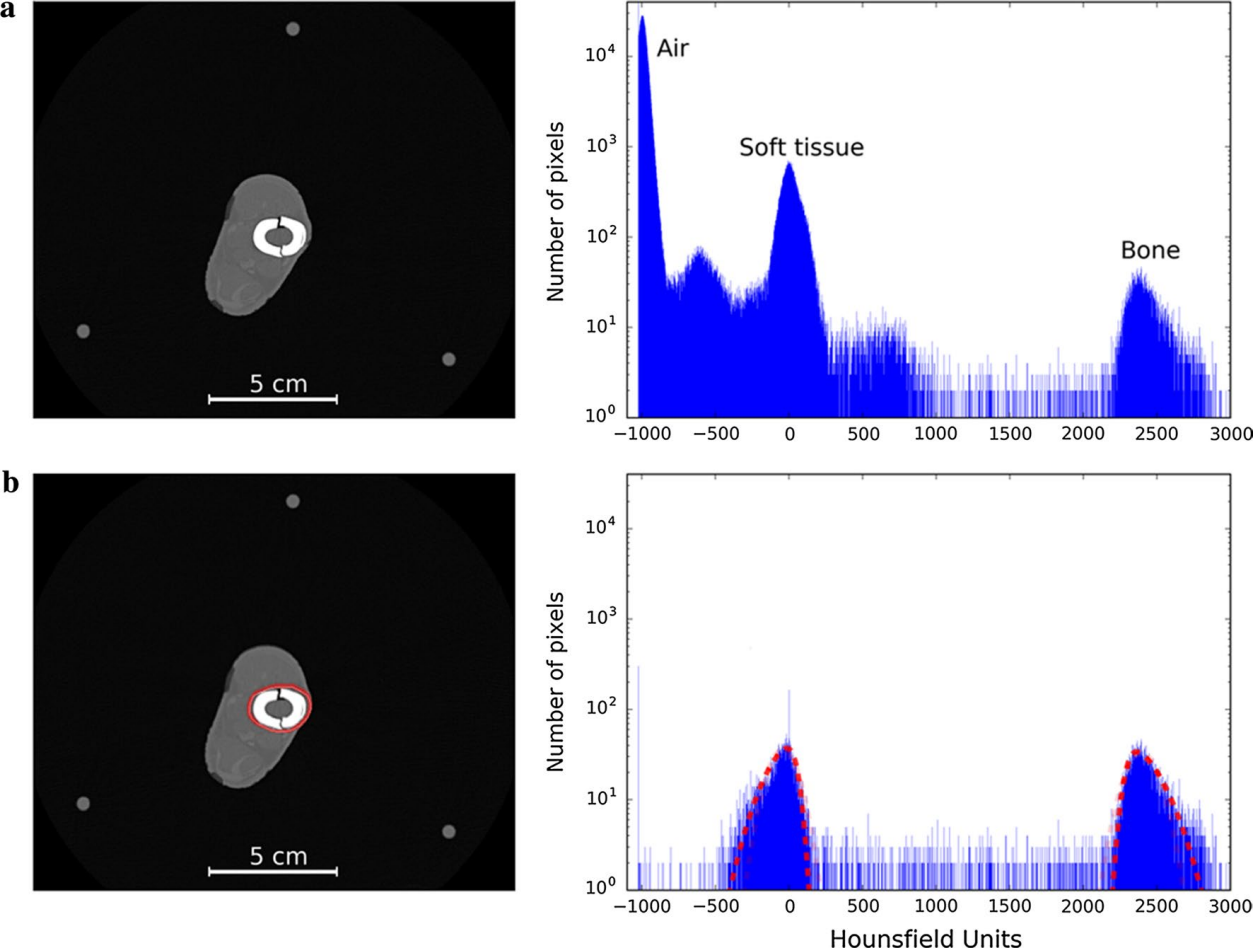
Example of information used for the analyses. **a** 2D axial slice (left) and histogram of whole image (right), with the three peaks representing air (black), soft tissue (grey), and bone (white). **b** 2D axial slice showing the region of interest circled in red (left) and histogram of the pixels inside the chosen area (right), where mean HU value and width are determined for bone and soft tissue using Gaussian fitting, shown in dotted lines

For the purposes of this study, the pixel values are displayed in two distinct ways: a traditional axial 2D image and a histogram. For the 2D image, the Hounsfield units are displayed as a greyscale, with negative values being increasingly dark and positive values being increasingly white (Fig. 3a, left). We used display settings with a centre of 300 HU and a window width of 2800 HU, which is not a typically used window width, but allows us to better observe the metal artefacts affecting the background of the images. The histogram is a graphical representation of the number of pixels of particular HU that are present in the field of view (Fig. 3a, right). To make the histogram more specific, a ROI around the tibia was defined (Fig. 3b, left); this removes the peak produced by air, making it easier to appreciate any change in the other two peaks (Fig. 3b, right). Whilst the histogram gives no spatial appreciation of individual pixels, it allows a quantitative analysis of the effect of the metal artefact on image quality by using Gaussian fitting of the bone and soft tissue peaks (Fig. 3b; dashed line; note that a Gaussian function appears as an inverted parabola on a logarithmic scale). The parameters of the peak that were analysed for the Gaussian functions were the mean HU value and the width (2.35 times the standard deviation).

## Results

Results are displayed as 2D images and ROI histogram, using the scale shown in Fig. 3. The ROI varies according to the slice being analysed, with the shape of an approximately elliptical shape that follows the outer boundary of the bone. Unless otherwise mentioned, the images show scans performed with single-energy CT.

### Baseline image

**Fig. 4:**
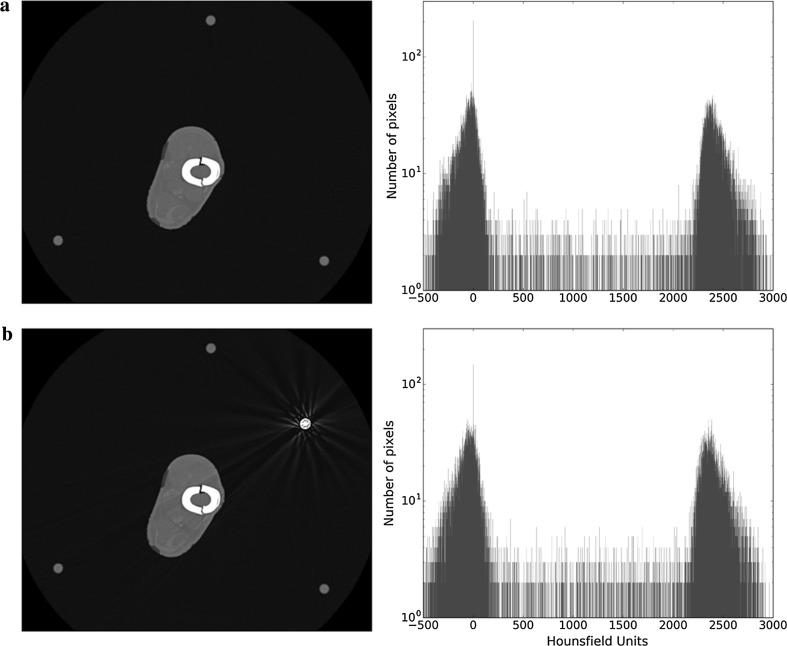
**a** Axial slice and histogram of the fracture site when there are not metallic components present. **b** Axial slice and histogram of the fracture site when only one rod is present

Figure 4a shows the 2D image and ROI histogram of an axial slice through the fracture site in a construct where there is no metal between the rings (although the plastic rods are evident). It represents the best quality image that can be obtained and therefore serves as the baseline for comparison with all subsequent images, where the presence of varying degrees of metal produces some degree of image degradation.

### Ilizarov frame

The presence of a single metal rod (Fig. 4b) causes fine streaks artefacts to radiate from it, but they have little effect on the quality of the image. On the histogram, the bone and soft tissue peaks are similar to those obtained from the image without rods.

**Fig. 5:**
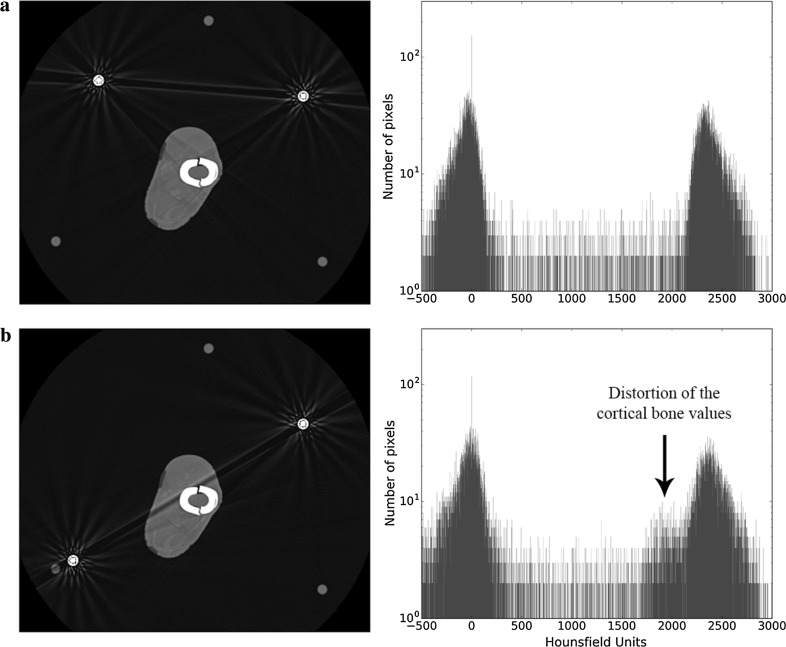
Axial slice and histogram of the fracture site when there are two metal rods. **a** The rods are located at the same side of the leg. **b** The rods are located at opposite sides of the leg. The rod coupling goes through the ROI

When two rods are used, the effect on the region of interest is highly dependent upon where they are placed (Fig. 5). On the 2D image it can be seen that the rods act as a couple, producing a broader and more noticeable streak that runs between them. This streak has a dark centre and bright edges. Where this streak crosses a part of the image that is already dark (Fig. 5a), its effect is negligible. However, if it crosses the region of interest, then its effect on detail is more pronounced (Fig. 5b). This can be appreciated on the histogram as a change in the spikes (wider bases and a change in the mean values).

**Fig. 6:**
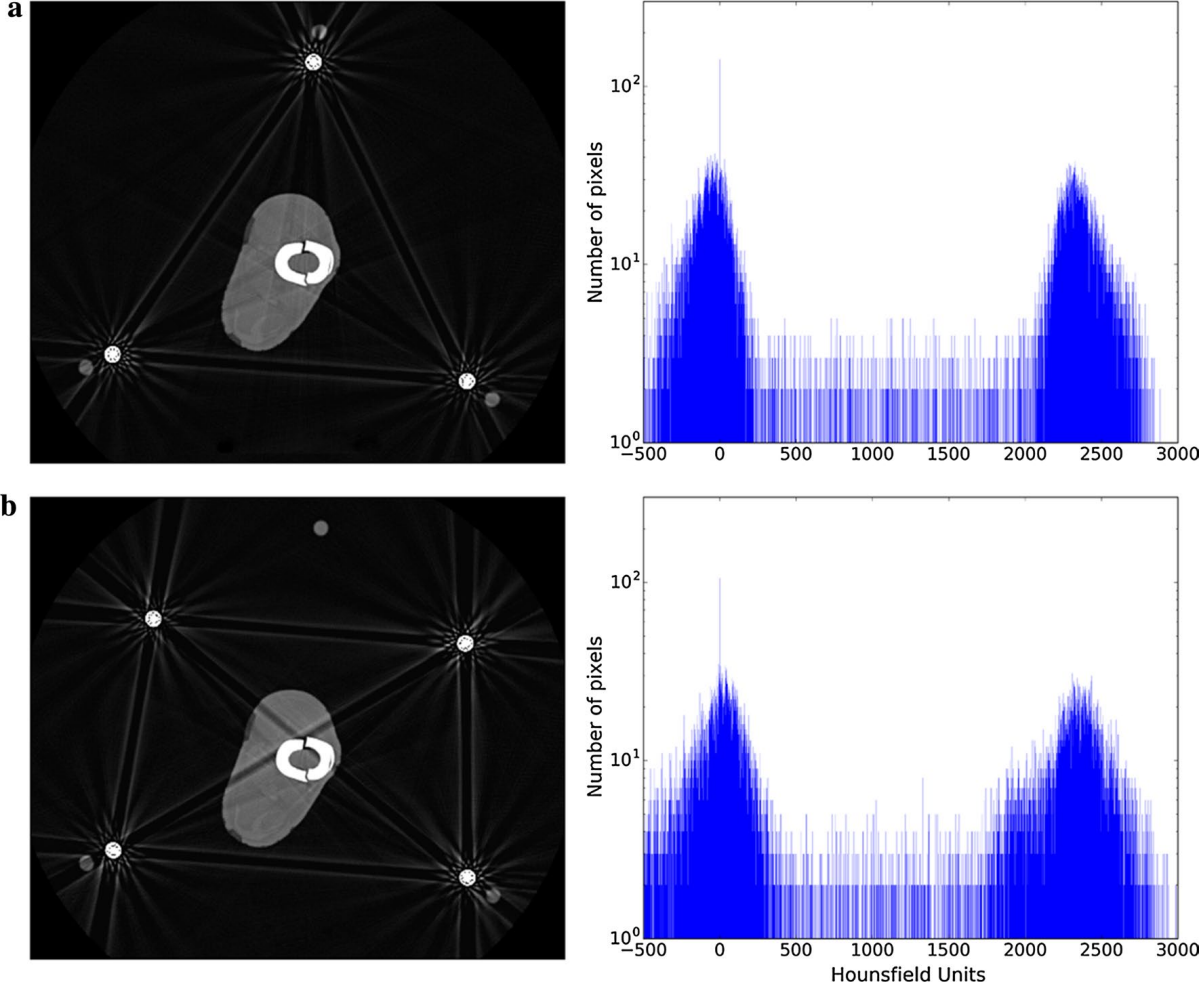
a Axial slice and histogram of the fracture site when there are three metal rods present. **b** Axial slice and histogram of the fracture site in the presence of four metal rods

Interestingly, when 3 rods are used, the effect on the region of interest is minimal (Fig. 6a); broad dark streaks with bright edges still occur between rods, but because they do not cross the ROI, they have little adverse effect. For the four-rod construct there are six such streaks, but only two of them cross the region of interest (Fig. 6b). The histogram shows a change in the spikes (wider bases), indicating loss of contrast and therefore image quality.

### Taylor Spatial Frame

**Fig. 7:**
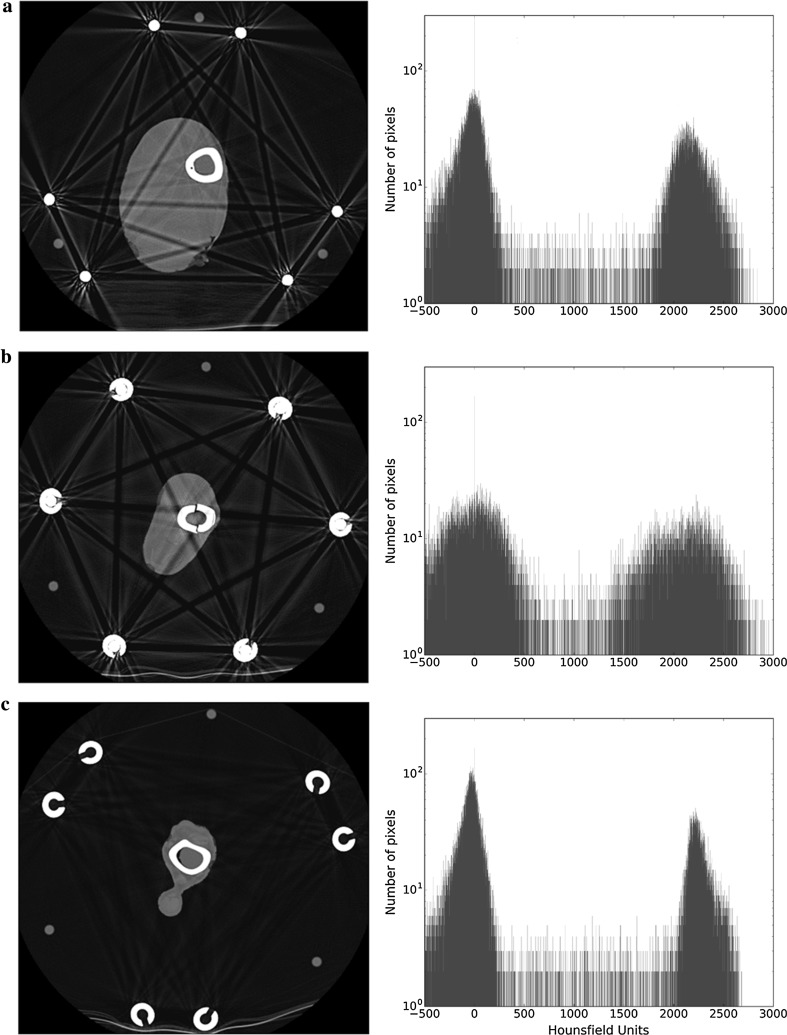
Axial slices and histograms of the leg when using the TSF. **a** Slice proximal to fracture site, with only the solid rods of the strut, can be observed. **b** Slice of the fracture site, showing a mixture of the solid rod and the tubular casing. **c** Slice distal to the fracture site, where only the tubular casing of the struts is observed

Geometric shape: The oblique orientation of the struts in the frame results in a distinctive geometric pattern of broad streaks, the nature of which differs according to the level scanned (Fig. 7). There are slices where the ROI is not directly affected by the streak (Fig. 7a), whilst in others, it is crossed several times (Fig. 7b).

Strut material: The makeup of the strut differs along its length, with one end being tubular casing (Fig. 7c) and the other end being a solid rod (Fig. 7a). In the middle, these two materials overlap to some degree (Fig. 7b). The severity of the broad streaks depends on which part of the strut is present in the particular slice.

### Dual-energy scanning/metal artefact reduction software

**Fig. 8:**
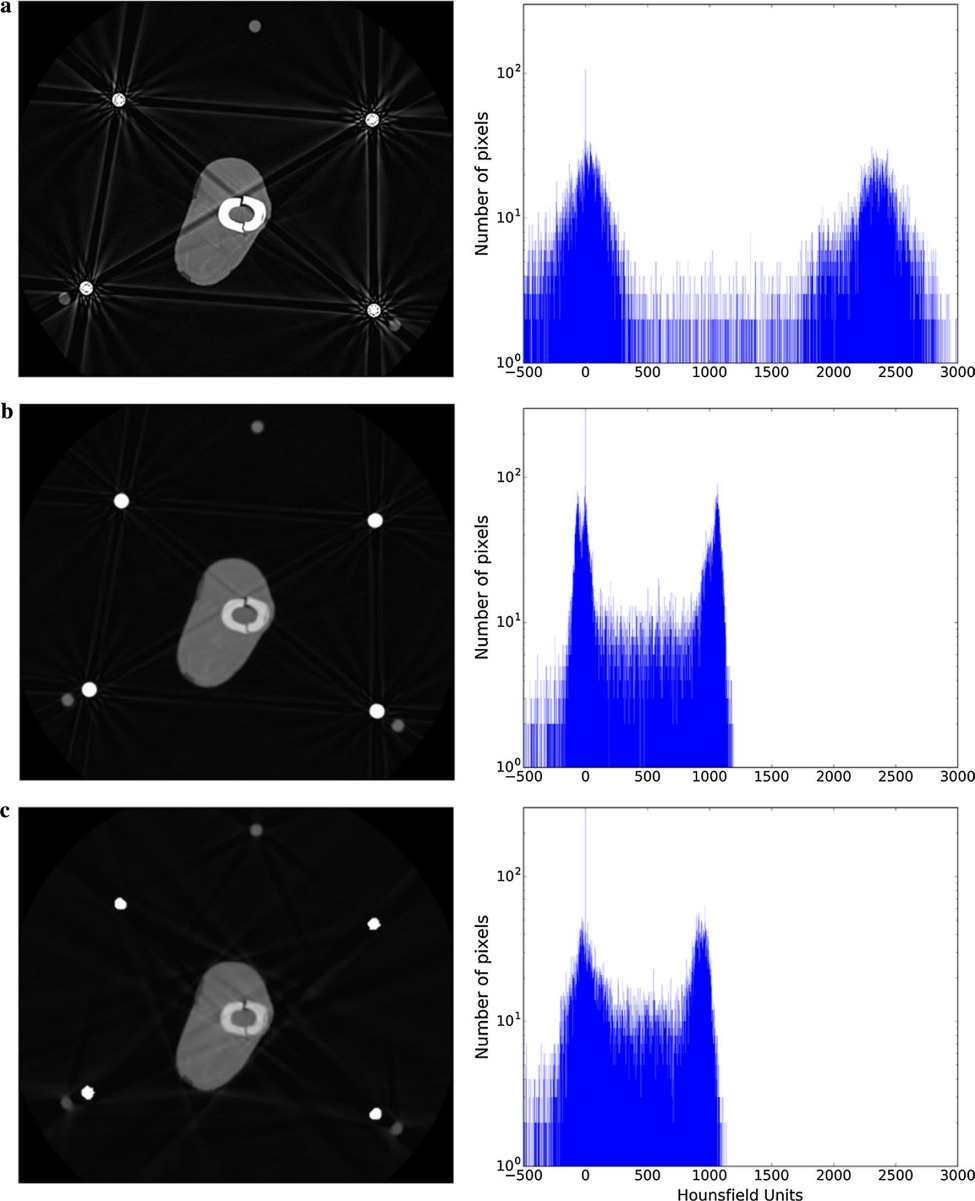
Effect of metal artefact reduction techniques in image clarity in the presence of 4 metal rods. **a** Single-energy scan. **b** DECT. **c** DECT + MARS. The bone peak moves to the left for DECT and DECT + MARS, which causes this tissue to become darker in the images when the window is conserved

**Fig. 9:**
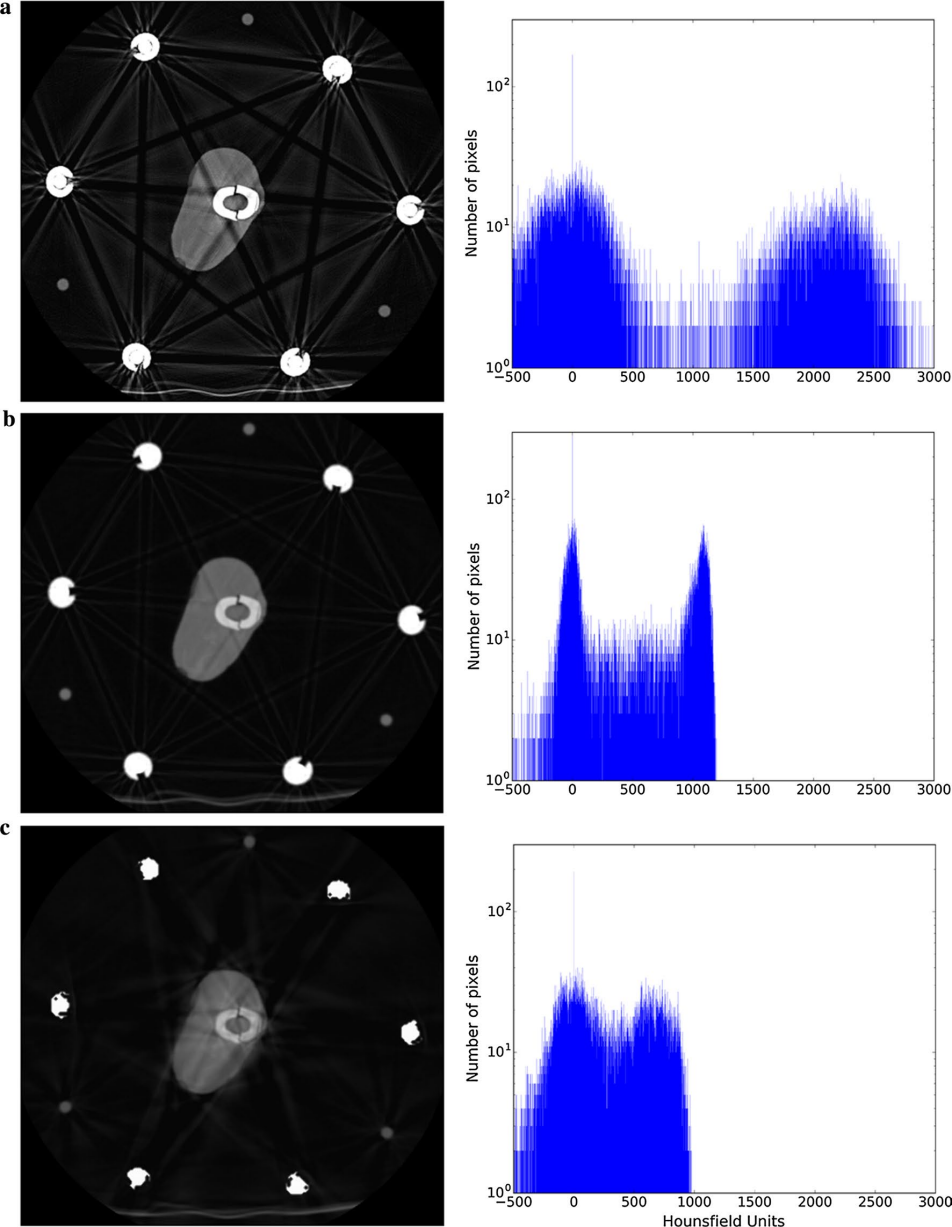
Effect of metal artefact reduction techniques in image clarity in the presence of the TSF. **a** Single-energy scan. **b** DECT. **c** DECT + MARS

Figures 8 and 9 show the effect of techniques used to reduce metal artefacts with the four-rod Ilizarov and TSF constructs, respectively. Figures 8a and 9a present the single-energy scans shown previously, whilst Figs. 8b and 9b are scans performed using DECT, and Figs. 8c and 9c present scans using DECT and MARS. Both of these modalities are associated with a decrease in the overall clarity of the image, particularly so with the MARS. For the histograms, the higher-energy scan has altered the HU value for bone from its mean of 2400 on the single-energy scan to around 1100, whilst the addition of MARS further results in the peaks tending to merge into one.

## Discussion

Obtaining good-quality images is a key part of the decision-making process for patients with circular external fixation. Frequently, nearby metal can obscure or have an adverse effect on details of the images obtained with plain film radiography and CT [3, 8].

The idea for this study came about because it is the practice of the senior author (PCH) to remove as many rods as possible (or exchange struts for rods) when CT imaging. Typically, this is done whilst the patient is lying on the CT bed, so that the temporarily weakened frame is not subjected to undue forces. The rods are then reinserted after the scan has been performed and before the patient gets off the bed. Since the scan itself is relatively quick, the surgeon may end up being present during the whole process of removal, scanning, and reattachment. It requires that the scan is performed at a time when the surgeon is available and is clearly time-consuming, for both the surgeon and the CT department. It therefore raises the question of how much benefit is derived by such a practice; is the improvement in image quality really worth all that effort? The aim of our study was therefore to investigate how the presence of rods/struts affects image quality. We chose a sheep leg over a human subject in order to scan it as many times as needed without concern for the adverse effects that ionising radiation can have on living humans.

**Fig. 10:**
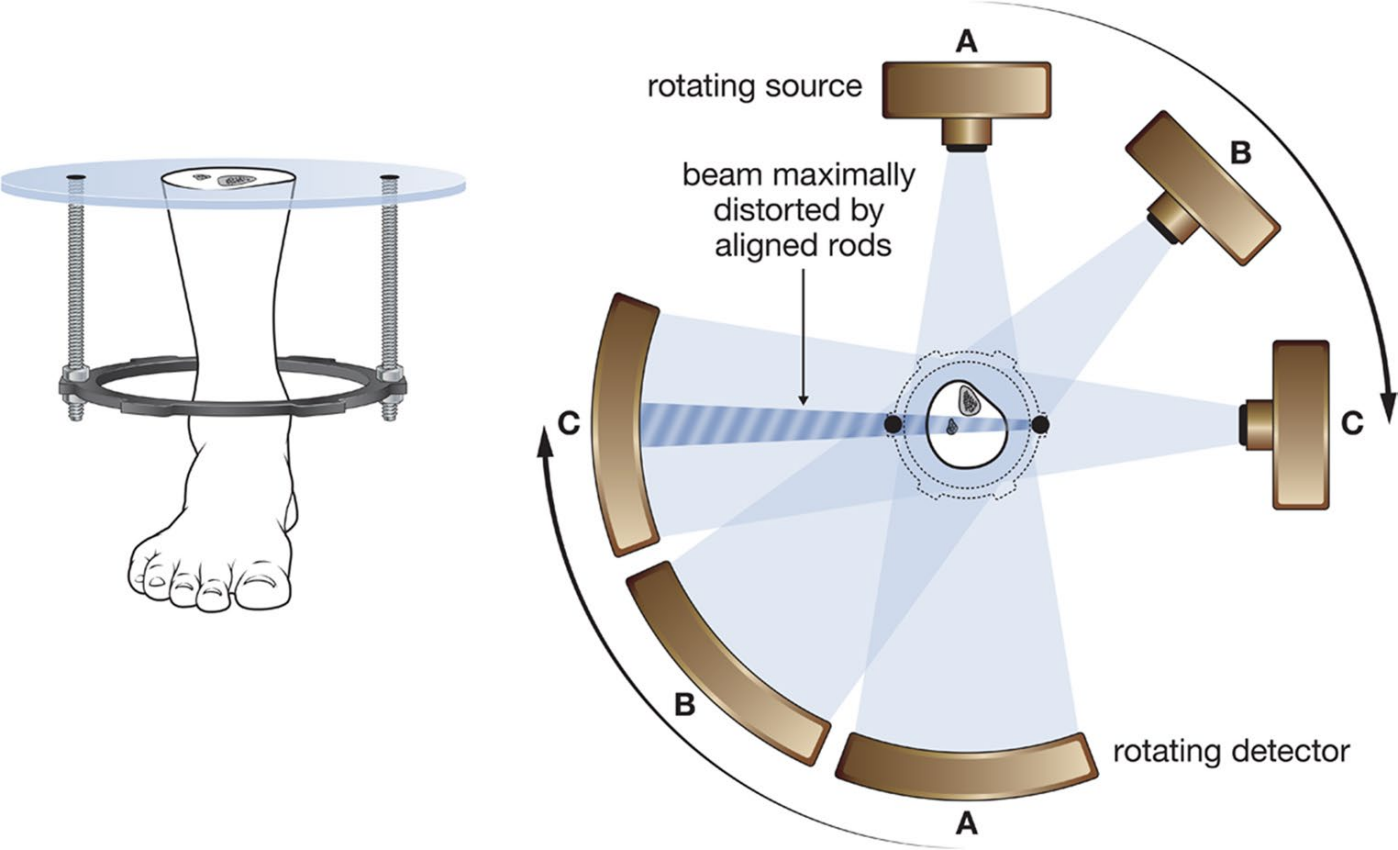
Helical nature of the CT scanning process, with X-ray source and detectors shown in three positions A–C. When the source completes a full rotation, a reconstruction algorithm converts the information to a 2D image slice. These slices can be displayed individually or assembled together to generate a 3D volume of the patient

The degradation of the CT image arises from the interaction between the poly-energetic X-ray beam and dense structures [4], creating two distinctive effects. The first effect (generalised noise proportional in degree to the overall amount of metal present on the axial slice) is seen as fine dark and bright streaks on the image [9, 10]. In this case, the artefact is distributed fairly proportionately throughout the image. The second effect, which is due to a pairing between rods and struts, causes a more noticeable broad dark streak with surrounding bright edges, and is a function of the helical manner in which the scan is acquired (Fig. 10). Whilst the general existence of artefacts from geometric considerations has been previously acknowledged [8, 11], our study focuses on its relevance for circular external fixation, for which we have coined the term *rod coupling*. Our study demonstrates that, in the case of circular external fixation, it is rod coupling that is the main factor causing degradation of image quality. The histograms show how changes in the values of bone and soft tissue due to the metal artefacts affect the contrast of the image. The spreading of the peaks in the histograms, which corresponds to bone and soft tissue, in general causes a loss of visibility of the trabecular structure which lays between the peaks, and therefore loses definition.

Whilst we acknowledge that mechanical reasons predominantly dictate the rod/strut configurations when building a circular frame, the effect of rod coupling during CT imaging can be minimised. Firstly, all or all but one rod can be removed during imaging; we see this as the ideal (Fig. 4), as in these cases there is no rod coupling. If this is not a possibility at that particular time, the second option is to configure the rods in a way that minimises the effect of the rod coupling on the area of interest. It can be appreciated in Fig. 6 that in the case of a four-rod construct, simply switching the two anterior rods to a more central single one will achieve this goal without particularly jeopardising mechanical strength for a short period. In the case of hexapod frames, whilst temporarily switching struts for rods would also appear to be a good idea, this can be more time-consuming, as often with hexapods the rings are not parallel, and therefore requiring the use of dished (conical) washers or the construction of hinges.

Can the CT technician configure the scan in a way that negates the need to remove or reconfigure rods/struts? In DECT, scans are acquired using two different energy settings and then synthesising pseudo-monochromatic scans at a variety of energies [12, 13]. The advantage of pseudo-monochromatic reconstructions is that the effect of streaking due to beam hardening, typically seen in CT imaging, is greatly reduced. Whilst the higher-energy setting reduces photon starvation and beam hardening, it results in some loss of detail (analogous to an over-penetrated plain X-ray). MARS works by evening out unexpected variation in pixels, but our study suggests that the image it produces compares poorly with the original; the mean HU values change considerably.

One limitation of our study relates to assessment of the quality of an image. Image quality is determined by, amongst other things, the resolution and the contrast. In this paper, our assessment/comparison of the quality of the 2D images is purely subjective. Whilst efforts are being made to produce an objective scoring system for the quality of digital images in general [14–16], to our knowledge no such score currently exists for radiography. By making our results section predominantly a display of images, we have allowed the reader to draw their own conclusions about the effects of the various constructs and metal artefact reduction modalities on image quality. Whilst Gaussian-fitting analysis of the histograms produces some degree of quantitative data analysis, it is a relatively crude measure of contrast and is not useful in clinical orthopaedic practice. For readers that are familiar with the attributes of Gaussian peak fitting, the quantitative parameters extracted from the peak-fitting procedure are displayed in Tables 1, 2, 3, and 4. The general observation is that as the peaks get broader and closer together, the ability to resolve features relating to those peaks diminishes. It can be seen that for Figs. 8 and 9, the data in Tables 3 and 4 demonstrate that the application of MARS reduces the level of artefacts but also affects the separability between the peaks.

**Table 1:** Mean and width values for soft tissue and bone peaks as calculated from the histograms when the number of metal rods is changed in single-energy scans

Number of rods/figure number	Soft tissue		Bone	
	Mean (HU)	Width (HU)	Mean (HU)	Width (HU)
0/Figure 4a	− 38	207	2396	251
1/Figure 4b	− 48	237	2377	280
2 (coupling outside ROI)/Fig. 5a	− 44	244	2355	298
2 (coupling crossing ROI)/Fig. 5b	− 23	284	2382	388
3/Figure 6a	− 50	284	2337	322
4/Figure 6b	16	367	2344	505

**Table 2:** Mean HU and width values for soft tissue and bone peaks as calculated from the histograms of different slices of the TSF single-energy CT scan

TSF Figure number	Soft tissue		Bone	
	Mean (HU)	Width (HU)	Mean (HU)	Width (HU)
Figure 7a	− 18	256	2160	416
Figure 7b	− 13	682	2111	846
Figure 7c	− 33	190	2231	212

**Table 3:** Mean HU and width values for soft tissue and bone peaks as calculated from the histograms of four-rod Ilizarov construct scans with single-energy CT, DECT, and DECT + MARS

Ilizarov (4 rods) Figure number/modality	Soft tissue		Bone	
	Mean (HU)	Width (HU)	Mean (HU)	Width (HU)
Figure 8a/single energy	16	367	2344	505
Figure 8b/DECT	− 22	132	1040	141
Figure 8c/DECT + MARS	5	284	922	181

**Table 4:** Mean HU and width values for soft tissue and bone peaks as calculated from the histograms of TSF construct scans with single-energy CT, DECT, and DECT + MARS

TSF Figure number/modality	Soft tissue		Bone	
	Mean (HU)	Width (HU)	Mean (HU)	Width (HU)
Figure 9a/single energy	− 13	682	2111	846
Figure 9b/DECT	− 4	125	1067	160
Figure 9c/DECT + MARS	13	430	618	486

From our perspective, the key point of this study is an appreciation of the phenomenon of rod coupling. Before CT scanning a limb with a circular external fixator, a thoughtful analysis should be made as to the selection of which rods to remove, or if they cannot be removed, how to configure them to avoid the worst effect of the metal artefacts on the region of interest. In our opinion, the best option is to temporarily reduce the number of rods to the bare minimum and/or to avoid rods at opposite sides of the ROI. That way, the surgeon can optimise image quality in the ROI, making it potentially easier to take medical decisions that impact the treatment and/or recovery of the patient.

### Removal of rods

**Fig. 11:**
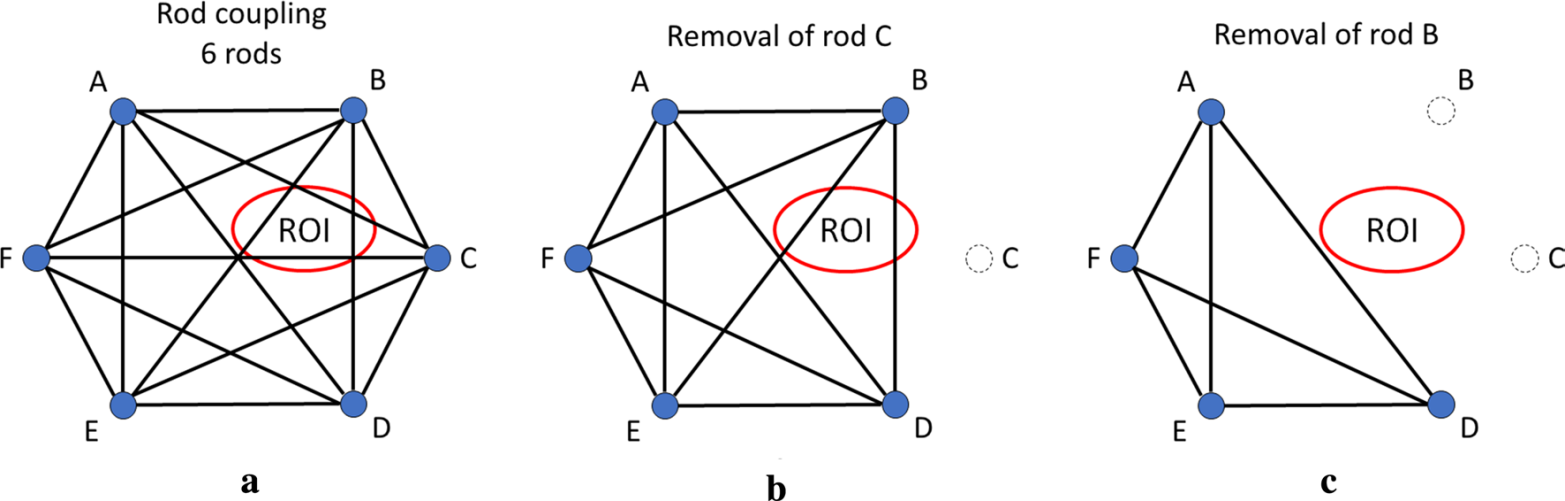
Schematic representation of rod coupling in the presence of 6 rods and the effect of rod removal on the metal artefacts. **a** Complete graph. **b** Resulting coupling after removal of rod C. **c** Resulting coupling after the removal of rods B and C

A rod-coupling line goes straight from every rod to every other rod (Fig. 11a; case with six rods). When a rod is removed, all lines connected to it are also deleted. One wishes to remove as many rods as are needed, to not have any rod-coupling lines going through the ROI. For example, if the maximum number of rods that can be left remaining is 3, then one could remove B + D + F, so as to have no rod couplings going through the ROI. A systematic process would be as follows:

1. Identify the rod(s) with the greatest number of lines passing through the ROI. In Fig. 11, B and C are the only rods with more than one line passing through the ROI. So, rod C is removed (Fig. 11b).
2. Again, we identify the rod(s) with the greatest number of lines passing through the ROI. Removing rod B will reduce the number of rod couplings in the ROI by two (Fig. 11c). If more than one rod reduces the number of rod couplings in the ROI by the same amount, a different criterion can be chosen, for example, if a more rigid frame can be obtained by removing one and not the other.
